# Above and beyond: biofilm and the ongoing search for strategies to reduce ventilator-associated pneumonia (VAP)

**DOI:** 10.1186/s13054-020-03234-5

**Published:** 2020-08-18

**Authors:** Takashi Sakano, Edward A. Bittner, Marvin G. Chang, Lorenzo Berra

**Affiliations:** 1 Division of Critical Care, Department of Anesthesia, Critical Care, and Pain Medicine, Massachusetts General Hospital, Boston, MA USA; 2 Division of Cardiac Anesthesia and Critical Care, Department of Anesthesia, Critical Care, and Pain Medicine, Massachusetts General Hospital, Boston, MA USA

**Keywords:** COVID-19, Coronavirus, Ventilator-associated pneumonia, VAP, Intubation, Biofilm, Endotracheal tube design, Coated endotracheal tubes

We read with great interest the article by Thorarinsdottir et al. [1] that compared biofilm formation on three endotracheal tube (ETT) types with the finding that biofilm formation was reduced in silicone and noble-metal coated ETTs compared to uncoated ETTs. Their findings have significant implications during the current pandemic given the prolonged intubation times of COVID-19 patients and many develop superimposed pneumonias during their hospital course. It is intriguing that simply changing the ETT’s coating may have significant implications in this patient population with already limited pulmonary reserve that is unable to tolerate additional insults to their lung from a ventilator-associated pneumonia (VAP). However, given the prolonged intubation times of COVID-19 patients, it is likely that the ETT biofilm burden will be substantial no matter which surface coating is utilized. Here, we discuss other strategies to reduce the incidence of VAP.

It is widely accepted that two mechanisms lead to VAP: aspiration of oral-gastric contents and microbial biofilm development on the ETT. Prevention is further complicated by a reduction in host mechanisms such as coughing and mucociliary clearance. Biofilms specifically cause two primary issues: (1) non-microbiological problem of intraluminal narrowing and (2) microbiological problem of biofilm development on the inner/outer surfaces of the ETT. The latter issue relates to our current discussion here.

Table 1 summarizes the mechanism, preventive measures, and methods that have been investigated to prevent aspiration and minimize biofilm burden related to tracheal seeding, microaspiration, biofilm formation, ciliary dysfunction, and the cough reflex. Despite substantial research that has continued, available tools to prevent VAP have changed very little. Many of the recommendations from the most recent 2014 Society for Healthcare Epidemiology of America (SHEA) and Infectious Diseases Society of America (IDSA) [2] consensus recommendations aimed at preventing VAP are controversial and have been questioned, including the efficacy and safety of oral chlorhexidine and head of bed elevation [3, 4].

**Table 1:** Investigated preventative measures and methods to reduce ventilator-associated pneumonia targeting mechanisms related to tracheal seeding, microaspiration, biofilm formation, ciliary dysfunction, and cough reflex

Source	Tracheal seeding	Microaspiration	Biofilm formation	Ciliary dysfunction	Cough reflex
**Mechanism**	Intubation advances oral and gastric contents into trachea	ETTs stent vocal cords open and oral secretions flow to distal airways by trickling through creases in ETT cuff	Biofilm embedded microbes are sources of embolic fragments that migrate to distal airway divisions	Low airway moisture causes ciliary dyskinesia, disrupting mucociliary clearance	ETT and sedation interfere with coughing and the cough reflex necessary for secretion clearance
**Prevention**	Suction and avoid advancing debris through glottis	Maintain cuff pressure of 20–30 cm H2O	Avoid unnecessary suctioning and bronchoscopy	Ventilate with warmed, humidified gas	Extubate early and perform frequent interruptions in sedation
**Methods investigated**	۰Oral/dental care ۰Preintubation oral antiseptics	۰ Novel cuff technologies and materials ۰ Continuous pressure cuff monitoring ۰ Subglottic secretions drainage ۰ Body position (lateral decub, prone, semi recumbent)	۰ Antimicrobial/antiseptic coated ETT ۰ Antibiotic impregnated ETT ۰ ETT cleaning devices	۰ Airway humidification ۰ Cuffless ETT with novel sealing	۰ Artificial cough maneuver

While it is important to develop technologies that minimize ETT microbial burden, other more practical, easily accessible, and affordable preventative measures should continue to be practiced and investigated. As previously advised by Klompas et al. [5], continuing with simple and proven methods that decrease aspiration load and biofilm formation should be maximized. While avoiding intubation may be the surest way to prevent VAP, this is not always possible. Incorporating the latest evidence into our current practice bundles may be key to improving clinical outcomes of COVID-19 patients.

## Data Availability

Not applicable.
